# Beyond T Cells: Understanding the Role of PD-1/PD-L1 in Tumor-Associated Macrophages

**DOI:** 10.1155/2019/1919082

**Published:** 2019-11-04

**Authors:** Di Lu, Zhen Ni, Xiguang Liu, Siyang Feng, Xiaoying Dong, Xiaoshun Shi, Jianxue Zhai, Shijie Mai, Jianjun Jiang, Zhizhi Wang, Hua Wu, Kaican Cai

**Affiliations:** Department of Thoracic Surgery, Nanfang Hospital, Southern Medical University, Guangzhou 510515, China

## Abstract

Programmed cell death protein 1 (PD-1) and its ligand PD-L1 have attracted wide attention from researchers in the field of immunotherapy. PD-1/PD-L1 have been shown to exist in many types of cells in addition to T lymphocytes, and studies have accordingly extended from their suppressive effect on T cell activation and function to examining their role in other cells. In this review, we summarize recent research on PD-1/PD-L1 in macrophages, with the aim of furthering our understanding of PD-1/PD-L1 and their detailed roles in macrophages. This information may provide additional insights for researchers, enrich the basic theory of anti-PD-1/PD-L1 immunotherapy, and thus ultimately benefit more patients.

## 1. Introduction

Programmed cell death protein 1 (PD-1) and its ligand PD-L1 have recently attracted much attention from researchers. Monoclonal antibodies targeting PD-1 or PD-L1, such as nivolumab, pembrolizumab, and atezolizumab, have been developed and entered into clinical trials. Furthermore, tumor immunotherapy based on PD-1/PD-L1 immune checkpoint blockade has demonstrated considerable efficacy in clinical trials in various cancers [1–6]. The PD-1 monoclonal antibody Opdivo (nivolumab) was approved by the US Food and Drug Administration in 2015 and was finally approved by the China Food and Drug Administration for the treatment of non-small-cell lung cancer in China on June 15, 2018, followed by another PD-1 monoclonal antibody, Keytruda (pembrolizumab), on July 26.

PD-1 was initially identified as a coinhibitory molecule on the surface of T lymphocytes. Interactions between PD-1 and its ligands, PD-L1 and PD-L2, activate the downstream signals of PD-1 and suppress T cell activation. CD8+ T cells are crucial for killing tumor cells, and their presence thus inhibits tumor elimination and allows tumor immune escape. Further research determined that PD-L1 was also widely located in hematopoietic cells, including T cells, B cells, dendritic cells, and macrophages, as well as in some nonhematopoietic cells such as vascular endothelial cells, astrocytes, and keratinocytes, while PD-L2 was expressed on macrophages, mast cells, and dendritic cells. Moreover, PD-L1 and PD-L2 were also expressed on tumor cells and tumor stroma cells [7], and PD-1 showed inducible expression on B cells, dendritic cells, and monocytes, as well as on T cells [8].

Macrophages are important immune cells that differentiate from monocytes, with roles in phagocytizing and killing pathogens, antigen processing and presentation, and cytokine secretion. Macrophages are commonly divided into M1 and M2 subsets, though some researchers have proposed the existence of more than two subsets [9]. M1 macrophages are considered to be “classic macrophages,” with roles in antigen presentation and proinflammatory cytokine secretion, while M2 macrophages are regarded as immunosuppressive “altered macrophages,” with functions in anti-inflammatory cytokine secretion and wound healing regulation [9]. Monocytes differentiate into different subsets of macrophages under the influence of different cytokines; for example, interferon- (IFN-) *γ*, lipopolysaccharide (LPS), and granulocyte-macrophage colony-stimulating factor cause monocytes to differentiate into M1 macrophages, while macrophage colony-stimulating factor, prostaglandin F, and vitamin D_3_ differentiate monocytes into M2 macrophages. M1 macrophages secrete proinflammatory cytokines such as interleukin- (IL-) 1*β*, IL-6, IL-12, IL-23, and tumor necrosis factor- (TNF-) *α*, and M2 macrophages secrete anti-inflammatory factors including IL-10, IL-13, and transforming growth factor- (TGF-) *β* and produce matrix metalloproteinase-2, arginase-1, and vascular endothelial growth factor-A [10, 11]. The respective cytokine secretions mean that M1 and M2 macrophages exert opposite functions. Numerous macrophages are present in tumors, with some tumor-associated macrophages (TAMs) resembling M1 and other M2 macrophages, while others appear to possess features of both. However, most TAMs appear and behave like M2-like cells [10], suggesting that macrophages could be polarized towards M2 inducibility in the tumor microenvironment, and that M2 macrophages may be an important factor in pretumorigenesis. However, M1-like macrophages occur during the initial phase of tumorigenesis, but are later transformed into M2-like cells, with an ultimate M2 predominance when the tumor metastasizes [11]. Macrophages thus apparently act as a “double-edged sword” in tumors, which appear to demonstrate an antitumor M1 phenotype but also a protumor M2 phenotype, with the ability to transform between phenotypes. Controlling this balance is therefore crucial to combatting cancer.

PD-1/PD-L1 is a notable immune checkpoint leading to T cell anergy. As noted above, PD-L1 is expressed in many cells while PD-1 is located on B cells, dendritic cells, macrophages, and T cells. It is therefore necessary to understand the functions of PD-1/PD-L1 in these cells and to clarify the similarities and differences in PD-1/PD-L1 between T cells and other cells. In this review, we focused on studies of PD-1/PD-L1 in macrophages.

### 1.1. PD-L1 in Macrophages

PD-L1 is widely expressed in a variety of cells, including antigen-presenting macrophages. Several studies have investigated the association between PD-L1 expression on macrophages and prognosis in cancer patients. A study of primary testicular lymphoma found that the number of PD-L1+ CD68+ macrophages was positively correlated with the number of PD-1+ T cells in the tumor, and that patients with high levels of PD-L1+ CD68+ macrophages or PD-1+ T cell infiltration showed favorable survival [12]. Another study in patients with hepatocellular carcinoma found similar results and showed that patients with PD-L1+ intratumoral macrophages had better survival than PD-L1− patients [13]. In contrast, however, a study of patients with stage I non-small-cell lung cancer found that patients with <6.3% intratumoral PD-L1+ macrophages experienced better survival than those with >6.3% intratumoral PD-L1+ macrophages [14]. The relationship between PD-L1 expression in intratumoral macrophages and prognosis in cancer patients thus remains controversial, and differences in tumor origin, PD-L1 positivity, and experimental methods may have contributed to the apparently conflicting results. Further studies using a formulated PD-L1+ value and accounting for tumor origin and experimental processes are therefore needed to resolve this relationship.

The function of PD-L1 in macrophages is also a matter of interest. As noted previously, the initial understanding was that PD-L1 combines with PD-1 in T cells to cause T cell dysfunction, with a similar function of PD-L1 in macrophages [15, 16]. The function of PD-L1/PD-1 binding may be considered to be the same in macrophages and T cells, given that it is a general ligand-receptor effect. However, Singhal et al. showed that PD-L1 expressed on macrophages did not inhibit the T cell response but merely protected macrophages from destruction by T cells, unlike PD-L1 expressed on tumor cells [17]. Since PD-L1/PD-1 is a couple of ligand-receptor, traditionally regarding PD-1 as receptor, it is likely to neglect the effect of PD-L1 as receptor on host macrophages after interaction with its ligand PD-1. One study showed that PD-L1 macrophages became larger and more active and their proliferation and survival abilities increased after treatment with PD-L1 antibodies. Macrophages treated with soluble CD80 (sCD80) or soluble PD-1 (sPD-1) were also larger and showed morphological changes and increased expression of CD86, major histocompatibility complex (MHC) II, and TNF-*α*, with sCD80 having a stronger effect than sPD-1. Moreover, PD-L1 signal suppressed the mTOR pathway to alter the transcriptome in macrophages [18]. These results indicated a regulatory role of PD-L1 in macrophage proliferation and activation. Another study showed that suppressing PD-L1 expression led to decreased expression of the M2 markers IL-10 and arginase-1, and increased expression of the M1 markers IL-12 and TNF-*α* in macrophages [19]. Similarly, Xiong et al. found that tumor macrophages treated with anti-PD-L1 showed decreased expression of arginase-1 and increased expression of inducible nitric oxide synthase (iNOS), MHC II, and CD40. These alterations, in addition to whole-transcriptome profiling, confirmed the transformation from immunosuppressive to immunostimulatory tumor macrophages [20], and implied a role for PD-L1 in M1/M2 polarization. These studies offer some information about the role of PD-L1 in macrophages and suggest that further studies should consider its function not only with regard to other cells but also its host cells. The above studies showed that PD-L1 was likely to be associated with M1/M2 polarization, leading to altered cytokine secretion and surface marker expression in macrophages. Further investigations of the mechanisms responsible for these alterations may clarify the role of PD-L1 in macrophages and help to establish a new theory regarding PD-1/PD-L1 immunotherapy.

Although the above studies have helped to reveal the function of PD-L1 in macrophages, its immunosuppressive function remains unclear. It is therefore necessary to identify the factors that regulate PD-L1 expression in macrophages. Some cytokines and exogenous macromolecules have been shown to regulate PD-L1 expression in macrophages. A study of gliomas showed that monocytes cultivated in glioma-conditioned medium expressed increased levels of PD-L1, which could be mitigated by IL-10 inhibition or IL-10 receptor inhibition. Furthermore, the production of IL-10 and expression of IL-10 receptor in monocytes was upregulated by culture them in glioma-conditioned medium, and PD-L1 expression was increased in monocytes treated with IL-10 [21]. This indicated that PD-L1 expression in macrophages could be regulated by IL-10 [22], while the secretion of IL-10 by macrophages could in turn be regulated by the tumor. Similarly, another study showed that tumors could induce monocytes to secrete TNF-*α* which could in turn upregulate PD-L1 expression in monocytes [23]. Another glioblastoma study showed that exosomes (extracellular vesicles) derived from glioblastoma stem cells induced M2 polarization and PD-L1 expression on monocytes [24]. Autophagosomes, another kind of extracellular vesicles released by tumor cells, were also reported to increase the expression of PD-L1 on macrophages in a Toll-like receptor (TLR4)-MyD88-p38-signal transducer and activator of transcription (STAT)3-dependent manner [25]. A lymphoma study reported that IL-27-induced upregulation of PD-L1 in macrophages could be mitigated by STAT3 inhibition, indicating that IL-27 may regulate PD-L1 expression in macrophages via STAT3 [26]. In addition, IL-4, IL-6, IL-10, and IL-13 could also increase PD-L1 expression in macrophages [27]. CXCL8, a chemokine predominantly secreted by macrophages and promoted by colony-stimulating factor-2, also induced PD-L1 expression on macrophages, while blocking CXCL8 decreased the proportion of PD-L1+ macrophages [28]. In addition to cytokines, pyruvate kinase isoform M2 (PKM2) may also be involved in regulating PD-L1 expression in macrophages. The PKM2 inhibitor TEPP-46 decreased LPS-induced PD-L1 expression in macrophages [29]. Furthermore, secreted phosphoprotein 1 (SPP1) also regulated PD-L1 expression in macrophages and affected macrophage polarization, and its knockdown led to decreased PD-L1 and M2 marker expression and increased M1 marker expression in THP-1 cells [19]. Moreover, the nuclear factor-*κ*B (NF-*κ*B) kinase inhibitor I*κ*K-16 decreased PD-L1 expression in macrophages, while IFN-*γ* increased PD-L1 expression and granulocyte-macrophage colony-stimulating factor had no significant effect on PD-L1 expression [30]. Mouse bone marrow cells, mostly macrophages and myeloid-derived suppressor cells, cocultured with bladder tumor cells strongly expressed PD-L1, as well as microsomal PGE2 synthase 1 (mPGES1), cyclooxygenase 2 (COX2), and prostaglandin E2 (PGE2), while inhibition of mPGES1 or COX2 led to decreased production of PGE2 and expression of PD-L1. These findings suggest that the COX2/mPGES1/PGE2 pathway may be involved in regulating PD-L1 expression in macrophages [31]. In addition, some biological macromolecules such as chitin have also been shown to regulate PD-L1 expression in macrophages [32]. Numerous studies have verified the high expression levels of PD-L1 in TAMs. The tumor-associated alterations in PD-L1 expression may be due to its secretion from tumor cells or from other cells, including macrophages. Tumor cells may release a substance or may induce other cells, including macrophages, to secrete a factor that in turn regulates PD-L1 expression in macrophages. This could be achieved via cell-to-cell contact. Tumor cells or tumor stroma may directly or indirectly alter PD-L1 expression in macrophages, thus creating an environment that favors tumor growth and leads to immune escape. Factors such as PGE2 and SPP1 could also regulate PD-L1 expression. However, further studies are needed to determine how these factors function and their relationship with tumors.

Interestingly, the above studies showed that PD-L1 in macrophages could lead to T cell anergy and M2 polarization, indicating that high levels of PD-L1 expression in macrophages were in accordance with an immunosuppressive tumor environment. Theoretically, a suppressive immune environment favors the proliferation and survival of tumor cells, leading to a poor prognosis. Conversely, however, several studies have shown that patients with high PD-L1 expression in macrophages had a better prognosis. A previously mentioned study of PD-L1 regulation of macrophage proliferation and activation [18] showed that macrophages with high PD-L1 expression had greater proliferation, survival, and activation abilities after treatment with anti-PD-L1 antibody, as well as increased expression of costimulatory molecules and cytokines. Macrophages treated with sPD-1 or sCD80 showed similar results, but sCD80 was more effective than sPD-1. These results suggest that anti-PD-L1 antibody had similar effects on PD-L1 to sCD80 or sPD-1, indicating that the PD-L1/PD-1 combination may not activate PD-L1 in macrophages, but may play a blocking role similar to anti-PD-L1 antibody. Alternatively, this combination may activate PD-L1 and then stimulate proliferation, survival, and activation pathways in macrophages, implying that the anti-PD-L1 antibody may function as an activator of PD-L1. CD80 is an alternative ligand for PD-L1, and CD80 had stronger effects on macrophages than PD-1. However, CD80 also acts as ligands for CD28 and CTLA4, both of which exist on T cells, and whether the effect of CD80 on macrophages can be attributed to the CD80/PD-L1 combination is thus unknown. Butte et al. proposed the specific combination between CD80 and PD-L1 and demonstrated the effect of this combination on T cells [33]. A more recent study found that the PD-L1/CD80 combination only occurred on one cell in cis-, but not on different cells in trans- [34]. Daisuke Sugiura's work reconfirmed this finding and further declared the significance of this combination that CD80 inhibit the effect of PD-1 activation by competing the binding site of PD-1/PD-L1 [35]. However, information on the PD-L1/CD80 combination and its effect in macrophages is currently lacking. Given that the interaction between pairs of PD-L1/PD-L2/PD-1, PD-L1/CD80, and CD80/CD28/CTLA4 is intricate and some molecules such as PD-L1 and CD80 can be expressed on both T cells and macrophages, the regulation between T cells and macrophages is complex. It is therefore necessary to take account of potentially confounding interactions between pairs of molecules other than PD-1/PD-L1 when analyzing the functions of PD-L1 in macrophages. The above studies did not assess the effects of CD80/PD-L1 and PD-1/PD-L1 combinations on macrophage proliferation, survival, and activation, and prognostic studies did not consider the influences of PD-1 and CD80. More studies are therefore needed to clarify the effect of PD-L1 on macrophages.

### 1.2. PD-1 in Macrophages

PD-1 was firstly discovered as a coinhibitory receptor on activated T cells. PD-1 activation resulted in phosphorylation of downstream molecules and attenuation of the activating signal from T cell antigen receptor (TCR) or CD28, thus inhibiting T cell activation [7]. Subsequent studies showed that, in addition to T cells, PD-1 was also expressed in B cells, NK cells, dendritic cells, monocytes, and macrophages [8, 36]. One study found that peritoneal macrophages in sepsis patients expressed high levels of PD-1, and that these cells were anergized and had lower bactericidal capacity. Furthermore, PD-1-/- mice with sepsis had lower mortality and a decreased bacterial burden compared with wild-type mice with sepsis [37]. These studies raise the question of the specific function of PD-1 in macrophages.

Although the function of PD-1 in inhibiting T cell activation is understood, T cells are adaptive immune cells and macrophages are innate immune cells, with differences in antigen recognition, activation, and effect. The function of PD-1 in macrophages may thus not be the same as that in T cells. The proportion of intratumoral PD-1+ macrophages in a colon cancer model was significantly higher than that in peripheral blood or spleen. The proportion of M2 macrophages among PD-1+ TAMs was also higher than that of M1 macrophages in a human specimen. In addition, PD-1+ macrophages expressed increased levels of CD206, CD11c, and CD4 and decreased MHC II, and showed weaker phagocytosis than PD-1− macrophages. Tumor progression was inhibited by treatment with anti-PD-1 or anti-PD-L1 antibody [36]. This study indicated that PD-1 plays a suppressive role in macrophages, inhibits macrophage phagocytosis, and may be associated with M2 polarization, while these effects may be reversed by anti-PD-1/PD-L1 antibody, with control of tumor progression. Another study of pulmonary metastasis of osteosarcoma showed similar results. Researchers found a high proportion of macrophages and NK cells in tumors expressing PD-1 in a mouse model, while anti-PD-1 therapy increased tumor infiltration of macrophages and NK cells and increased the proportion of M1 and decreased the proportion of M2 cells in the tumor. This study further confirmed that the effect was due to macrophages rather than NK cells by depleting each cell type, respectively [38]. In a study of spinal cord injury, PD-1 deficiency induced M1 polarization of macrophages, possibly via upregulated phosphorylation of STAT1 and NF-*κ*B and downregulated phosphorylation of STAT6 [39]. These studies revealed the association between PD-1 and M2 macrophages and noted an important role of macrophages in antitumor immunity. PD-1/PD-L1 immune checkpoint immunotherapy could potentially alter the function of tumor macrophages by affecting the M1/M2 polarization. It is therefore crucial to confirm the effect and mechanism of PD-1 in macrophage polarization, as a basis for exploring new viewpoints in PD-1/PD-L1 immunotherapy. In addition, PD-1 could also influence cytokine secretion by macrophages. In a study on hepatitis C virus, PD-1 expression was negatively correlated with IL-12 expression in monocytes in peripheral blood, and IL-12 expression fell in line with decreased STAT1 phosphorylation. However, anti-PD-1 antibody administration improved IL-12 production and STAT1 activation in macrophages. This study suggested that PD-1 activation may reduce IL-12 production in macrophages through decreasing STAT1 phosphorylation, and could be reversed by anti-PD-1 antibody [40]. Another study found that PD-1/PD-L1 blockade could promote IL-6 production by PD-1+ macrophages, while activation of PD-1 by recombinant PD-L1 reduced IL-6 production from macrophages, revealing the regulatory role of PD-1 on IL-6 production in macrophages [41]. The above studies show that PD-1 can regulate cytokine secretion from macrophages, which can further regulate the function of immune cells via the immunomodulatory role of cytokines, thus affecting tumor growth and progression. However, more studies are needed to clarify the complex relationship between PD-1 and cytokine production in macrophages.

Overall, PD-1 expressed on macrophages plays an immunosuppressive role in immunity. However, although increased macrophage expression of PD-1 is frequently observed in patients with diseases such as infections and cancer, the mechanisms regulating the expression of PD-1 remain unclear. One study indicated that constitutive and IFN-*α*-induced PD-1 expression in macrophages were attributed to interferon-sensitive responsive element (ISRE), STAT1, and STAT2, while JAK/STAT inhibition could reduce IFN-*α*-mediated PD-1 expression in macrophages [42]. Another experiment showed that LPS and zymosan promoted PD-1 expression in macrophages through TLR4 and TLR2, respectively, while poly(I:C)-activated TLR3 had no such effect. The crucial downstream molecule, NF-*κ*B, was studied in an attempt to understand the mechanism involved. LPS- or zymosan-stimulated macrophages treated with an NF-*κ*B inhibitor expressed less PD-1 than macrophages without NF-*κ*B inhibitor, suggesting that NF-*κ*B is a pivotal molecule in TLR-mediated PD-1 expression in macrophages [43]. These studies explored signaling pathways involved in regulating PD-1 expression in macrophages, and factors affecting these signaling molecules may thus also regulate PD-1 expression in macrophages.

**Figure 1:**
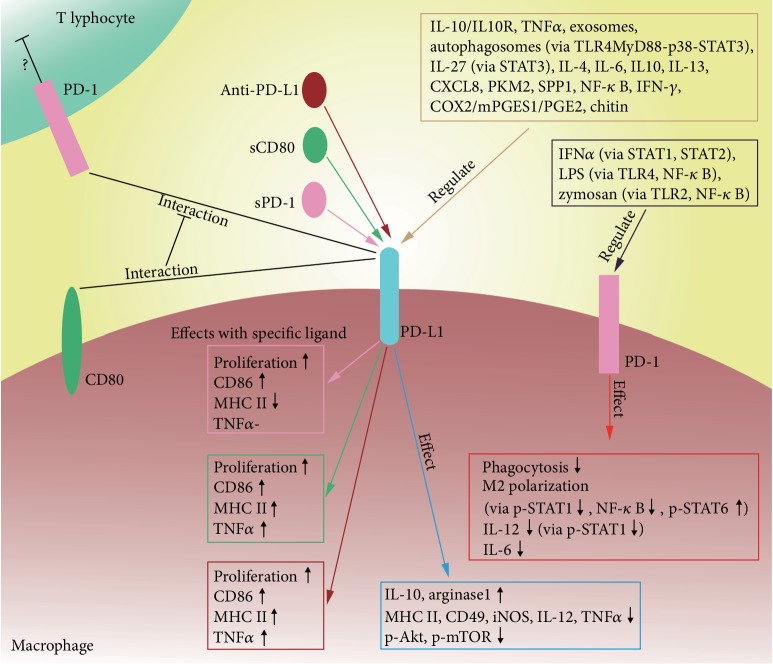
Summary for the regulation and function of PD-1 and PD-L1 in macrophages.

Further studies of PD-1 will extend our understanding of PD-1 from its role in T cells to many other cell types, including macrophages. In terms of antitumor immunity, the immune system is an integrated system involving immune cells and molecules, and their interactions. Therapy targeting a single molecule or cell may thus have little effect, while therapy targeting PD-1/PD-L1 has shown favorable results. Correspondingly, PD-1 was expressed not only in T cells but also in many other types of immune cells, which may help to explain the results of anti-PD-1/PD-L1 therapy, given that anti-PD-1/PD-L1 therapy may affect immune cells besides T cells. Further studies on PD-1 in these cells are required to clarify this issue. This review presented the current knowledge regarding the roles of PD-1 and PD-L1 in macrophages (Figure 1). However, information is limited, and more studies are needed to explore the functions of PD-1/PD-L1, the factors affecting their expression, their relationship with tumor progression, and their potential as a therapeutic target. One current idea in tumor immunotherapy involves transforming M2 into M1 macrophages. The proposed relationship between PD-L1/PD-1 and macrophage polarization suggests that PD-L1/PD-1 may be a potential target for achieving this objective, though more research is warranted to confirm this. Anti-CD47-signal regulatory protein alpha (SIRP*α*) immunotherapy is another possible avenue of research. A study of small-cell lung cancer demonstrated that CD47-blocking antibody or inactivation of the CD47 gene suppressed the growth of tumors with high expression of CD47. Blocking the CD47-SIRP*α* interaction by using an anti-CD47 antibody could abrogate the suppression of macrophage phagocytosis from CD47 on tumor cells [44]. In addition, combined anti-PD-1/PD-L1 and anti-CD47 therapy has also been proposed [45], and PD-L1 blockade was shown to improve the efficacy of CD47 antagonism [46]. It may therefore be beneficial to combine anti-PD-L1 and anti-CD47 therapies to activate the innate and adaptive immune systems simultaneously; however, the efficacy and safety of this approach needs to be tested.

## 2. Conclusions

Innate immunity is pivotal to the human immune system, in terms of initiating and determining the type and magnitude of adaptive immunity. As antigen-presenting cells, macrophages play a crucial role in innate immunity, with the M1 and M2 subsets showing nearly opposite functions. The well-known pair of molecules PD-1/PD-L1 is expressed on most immune cells, as well as tumor cells, and is also expressed on macrophages. Several studies have revealed the relationship between PD-1/PD-L1 and macrophage polarization, but the detailed role of PD-1/PD-L1 in macrophages is still unclear. Further studies are therefore needed to clarify many aspects of the roles of PD-1/PD-L1 in macrophages. Furthermore, the impacts of interfering with PD-1/PD-L1 on macrophages and new strategies to regulate macrophages in clinical practice also need to be explored. More work is therefore needed to provide a thorough understanding of the role of PD-1/PD-L1 in macrophages and to allow the development of therapeutic methods to benefit patients.
